# Role of Histone Deacetylase and Inhibitors in Cardiovascular Diseases

**DOI:** 10.1111/cpr.70077

**Published:** 2025-06-11

**Authors:** Li‐Ying Zhang, Yue‐Yue Wang, Ri Wen, Tie‐Ning Zhang, Ni Yang

**Affiliations:** ^1^ Department of Pediatrics, PICU Shengjing Hospital of China Medical University Shenyang China

**Keywords:** cardiovascular diseases, cell proliferation, histone deacetylase, mitochondrial metabolism

## Abstract

Histone deacetylase(HDAC) is Zn^2+^‐dependent histone deacetylases that regulate the key signalling pathways involved in gene transcription. 11 isoforms have been identified. Recent in vitro and in vivo studies have shown that HDACs are involved in the pathophysiology of cardiovascular diseases (CVDs) and play important roles in cell proliferation, differentiation and mitochondrial metabolism. In terms of physiological mechanisms, HDAC1–6 may play important roles in normal cardiac development and physiological function, while HDAC7 regulates angiogenesis. In pathological processes, class I HDACs function as pro‐hypertrophic mediators, whereas class II HDACs act as anti‐hypertrophic mediators. HDAC1–3, 6, 9, and 11 participate in lipid cell formation, oxidative stress and endothelial cell injury through multiple signalling pathways, contributing to the pathogenesis of atherosclerosis. In addition, HDACs also play a role in CVDs such as heart failure, myocardial fibrosis, pulmonary hypertension and diabetic cardiomyopathy. In view of this, we reviewed the regulatory pathways and molecular targets of HDACs in the pathogenesis of CVD. In addition, we summarise the current discovery of inhibitors targeting HDACs. HDAC inhibitors have shown promising therapeutic progress in animal experiments, but clinical trials to demonstrate their efficacy in humans are still lacking. A better understanding of the role of HDACs in CVD provides a new direction for the development of therapeutic interventions and holds significant research value.

## Graphical abstract

HDACs play an important role in the occurrence and development of cardiovascular diseases, such as myocardial hypertrophy, hypertension, and atherosclerosis. HDAC inhibitors have broad prospects for the treatment of CVD, and different HDAC subtypes can act through different mechanisms.

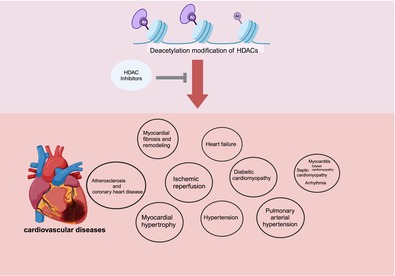

**Abbreviations**
AF: atrial fibrillation
Agt: angiotensinogen gene promoter
ANG II: angiotensin II
ANP: atrial natriuretic peptide
Arg2: Arginase 2
AS: atherosclerosis
BMP4: morphogenetic protein‐4
BNP: Brain natriuretic peptide
CAD: coronary artery disease
CG: CG200745
CHD: coronary heart disease
CHF: congestive heart failure
CK2α1: casein kinase‐2α1
COL1A2: collagen type I gene
CSEγ: endothelial cystathionine *γ*‐lyase
CVD: cardiovascular diseases
CycD1: cyclinD1
DCM: diabetic cardiomyopathy
DUSP5: dual‐specificity phosphatase 5
Egr‐1: early growth response protein‐1
EndMT: endothelial‐to‐mesenchymal transition
Endog: nuclease endonuclease G
eNOS: endothelial nitric oxide synthase
ERG: E26 transformation‐specific(ETS)‐related gene
ERK: extracellular signal‐regulated kinase
FDA: food and drug administration
FGF9: Fibroblast growth factor 9
FOX: forkhead box
GATA6: GATA‐binding factor 6
GSDMD: gasdermin D
HDAC: histone deacetylase HDAC
HFD: high‐fat diet
HFpEF: heart failure with preserved ejection fraction
Hsp70: Heat shock protein
I/R: ischemia/reperfusion
IGF: insulin‐likeGF2
IH: intermittent hypoxia
KCNN: Kpotassium voltage‐gated channel subfamily N
KLF4: Kruppel‐like factor 4
MAPK: mitogen‐activated protein kinase
MEF2: myocyte enhancer factor 2
MF: myocardial fibrosis
MGCD0103: mocetinostat
MKK3: mitogen‐activated protein kinase kinase 3
MMP‐9: matrix metalloproteinase‐9
NAD: nicotinamide adeninedinucleotide
Nappa: natriuretic peptide A
NET: neutrophil extracellular traps
NF‐κB: nuclear factor kappa‐light‐chain‐enhancer of activated B cells
NOX‐4: nicotinamide adenine dinucleotide phosphate oxidase 4
PAH: pulmonary arterial hypertension
PAR2: protease‐activated receptor 2
PI3K/Akt: phosphoinositide 3‐kinase and Protein Kinase B
PP2A: protein phosphatase 2A
PPAR‐*γ*
: peroxisome proliferator‐activated receptor
PTB: polypyrimidine tract‐binding protein
RASSF1A: Ras association domain family member 1A
RGFP966: research group for functional proteomics 966
RNase1: endonuclease ribonuclease 1
ROS: reactive oxygen species
SMAD: small mothers against the decapentaplegic
SOD‐1: superoxide dismutase −‐1
STAT3: signal transducer and activator of transcription 3
TAC: aorta coarctation
TIMP‐1: tissue inhibitor of metalloproteinase‐1
TNF‐α: tumourtumor necrosis factor‐alpha
Tregs: regulatory T cells
TSA: trichostatin A
TSC2: Tuberous sclerosis complex 2
VCAM‐1: vascular cell adhesion molecule
VPA: valproic acid
VSMC: vascular smooth muscle cell
*α*‐SMA: *α*‐smooth muscle actin
*β*‐AR: *β*‐adrenergic receptor

## 1 Introduction

The histone deacetylase (HDAC) family is a mammalian homologue of the yeast Rpd3/HDA1 superfamily, a conserved HDAC that is Zn^2+^‐dependent [1]. 11 members(HDAC1‐HDAC11) have been identified, with each isoform having a different sequence and length [2]. Class I HDACs are localised in the nucleus, Class IIb and Class IV mainly in the cytoplasm, and Class IIb shuttles between the nucleus and the cytoplasm depending on its phosphorylation state in response to cellular signals. Recent studies have shown that HDACs are involved in a variety of physiological and pathological processes, significantly influencing organ and tissue functions.HDACs are associated with many cellular phenotypes, including oxidative stress, autophagy, inflammation and gene transcription. They are implicated in mitochondrial biogenesis and metabolism [3, 4].

A recent epidemiological study found that the incidence of cardiovascular disease (CVD) in men and women was 52.6% and 57.2%, respectively, with the mortality rate exceeding 20% [5]. CVD has placed a significant burden on the healthcare system, and its incidence is increasing, exacerbated by suboptimal lifestyle and dietary habits. Although the molecular mechanism underlying CVD remains unclear, mitochondrial function, inflammatory response, oxidative stress, apoptosis and other processes are closely related to its pathogenesis. Notably, an increasing number of studies have linked HDAC with different pathological processes, suggesting that the HDAC protein family may play a key regulatory role in the occurrence and development of CVD, as well as a potential molecular therapeutic target [6].

In this review, we summarise the latest advancements regarding the HDAC protein family in cardiovascular physiology and pathology, discuss the mechanisms of action involved in the occurrence and development of CVD and summarise the clinical research status of HDAC inhibitors. Our analysis provides novel perspectives into the role of HDACs in the pathogenesis of CVD and provides potential opportunities for the prevention and treatment of CVD.

## 2 HDAC Family

HDAC is divided into 11 HDACs and 7 sirtuins, which mainly act on the core N‐terminal lysines of histones by removing acetyl groups from N‐terminal lysine residues. HDAC has zinc‐dependent activity, whereas sirtuins have nicotinamide adenine dinucleotide (NAD)^+^‐dependent activity. The classical HDAC family is divided into three classes: Class I HDAC (1,2,3,8), Class II HDAC (4–7,9,10) and Class IV HDAC11 [2]. The subcellular localisation of various HDAC cells differs; in general, Class I HDACs are mainly localised in the nucleus, IIa and IV are mainly localised in the cytoplasm [7, 8], while Class IIb shuttles between the nucleus and cytoplasm according to its phosphorylation state under the action of different proteases [9, 10, 11]. Class I HDAC is involved in cell death, fibroblast activation and proliferation, inflammatory response, oxidative stress and endothelial‐mesenchymal transition, and they play an important role in atherosclerosis(AS), ischemia/reperfusion (I/R) myocardial injury and myocardial fibrosis (MF). Because of their localisation in the nucleus, Class I HDACs regulate the transcription of multiple cardiovascular system genes. In addition, Class I HDACs serve as regulators of cardiac hypertrophy, while Class II HDACs also play a role in this process. The pro‐hypertrophic effect of Class II HDACs is closely related to the shuttling of HDAC4 and HDAC5 between the nucleus and cytoplasm, influencing the transcription of the myocyte enhancer factor 2 (MEF2) gene [9, 10, 11, 12]. After the phosphorylation of HDAC4 and HDAC5 and their subsequent nuclear export, the inhibition of MEF2 transcription is alleviated, leading to upregulation of MEF2 transcription that promotes hypertrophy. HDAC11, the only protein molecule in Class IV HDAC [7], can participate in AS through the pyroptosis pathway [13] and participates in the progression of diabetic cardiomyopathy (DCM) through mechanisms such as apoptosis, oxidative stress and inflammatory responses [14]. The classical HDAC family is involved in various CVD phenotypes through classical signalling pathways, including Phosphoinositide 3‐kinase and Protein Kinase B (PI3K/Akt), Mitogen‐Activated Protein Kinase (MAPK), Nuclear Factor kappa‐light‐chain‐enhancer of activated B cells (NF‐κB). In addition, the pharmacological effects of targeting HDAC are described in detail in the following sections.

## 3 Role of HDAC in Cardiac and Vascular Development

Histone acetylation is one of the most widely studied post‐translational protein modifications and is closely related to the regulation of gene expression and transcription during cardiovascular development.

HDAC1 suppresses early cardiac development while promoting late cardiac development. Early acetylation enhances the upregulation of islet‐1 and Nkx2 in cardiac progenitor cells, whereas late acetylation leads to the upregulation of octamer‐binding transcription factor 4. This results in the withdrawal of developmental pluripotency and the expression of cardiomyocyte‐specific contractile proteins [15]. The study by Trivedi et al. revealed that some HDAC2‐deficient mice died during the perinatal period and had marked cardiomyocyte defects and abnormal cardiomyocyte maturation [16]. Jang et al. found that HDAC3‐knockout (HDAC3‐KO) mice exhibit reduced epicardial cell proliferation [17]. The downregulation of microRNA (miR)‐322 and miR‐503 by HDAC3 leads to the upregulation of fibroblast growth factor 9 (FGF9) and Insulin‐like GF 2 (IGF2) expression [17]. Increased levels of FGF9 and IGF2 have been shown to stimulate the proliferation and growth of cardiomyocytes through their respective receptor interactions, whereas HDAC2 is essential for mouse heart development. HDAC4 and HDAC5 nuclear export promotes the transcription of related developmental factors and plays a role in cardiovascular development [18, 19]. The peptide ligand apelin binds to its receptor apelin receptor, leading to the activation of G protein α13, phosphorylation of HDAC4 and HDAC5, subsequent nuclear export, promotion of myocyte enhancer MEF2 expression and regulation of cardiovascular endothelial cell formation [20]. In addition, a study by Ye et al. revealed that HDAC5 is involved in the alternative splicing of MEF2a and MEF2b by Polypyrimidine tract‐binding protein (PTB) [21].

During the process of heart valve development, HDAC stimulates the expression of bone morphogenetic protein‐4 (BMP‐4) in atrioventricular septal cardiomyocytes via the Wnt/β‐catenin signalling pathway, thereby facilitating the epithelial–mesenchymal transition of endocardial cells [22].

HDAC3 is present in the myocardial sarcomeres and plays a role in myocardial contraction. Samant et al. identified HDAC3 in the sarcomeres of the myocardium, which can deacetylate myosin heavy chain (MHC) isoforms, thereby playing a role in myocardial contractility [23]. The presence of HDAC4 in myocardial sarcomeres does not exert any effect [24]. However, transgenic HDAC4 mice revealed cardiac dysfunction and defective vascular growth compared with wild‐type mice [25]. HDAC4 is an important regulator of cardiovascular development. HDAC6 was found to colocalise with the Z‐disk of sarcomeric proteins and regulate titin compliance through a reversible acetylation process, thereby modulating myofibril stiffness [26].

HDAC7 induces the differentiation of ESCs (embryonic stem cells) into smooth muscle cells by regulating the SRF–myocardin complex to mediate alternative splicing [27]. The spliced isoform of HDAC7 promotes vascular smooth muscle cell proliferation and neointimal formation by facilitating the nuclear translocation of β‐catenin [28]. Additionally, HDAC7 directly binds to β‐catenin, suppressing its activity, preventing its nuclear translocation and downregulating TGF‐β1 (transforming growth factor‐beta 1), Id2 (inhibitor of DNA binding 2) and cyclin D1, thereby inhibiting endothelial cell growth [29]. These findings suggest that HDAC7 plays a critical role in angiogenesis.

HDAC9 acts as a repressor of cardiac hypertrophy signalling and plays a central role in suppressing a subset of cardiac stress responses [30]. Additionally, it regulates redundant functions during cardiac development [31].

These findings suggest that HDAC1‐6 may play integral roles in normal cardiac development and physiological function. While their involvement in transcriptional regulation, cardiac development and myocardial contraction has been established, the precise molecular mechanisms require further investigation. HDAC7 has been demonstrated to play a critical role in endothelial cell differentiation and angiogenesis. The roles of HDAC8, HDAC10 and HDAC11 in cardiovascular development remain poorly understood. Current studies have only found that HDAC9 is a repressor of cardiac hypertrophy, but the specific physiological role and mechanism are not clear. Overall, there is a need to further explore the physiological role of HDACs during the development of the cardiovascular system.

## 4 Role of HDAC in CVD

### 4.1 Role of HDAC in Myocardial Hypertrophy

**FIGURE 1:**
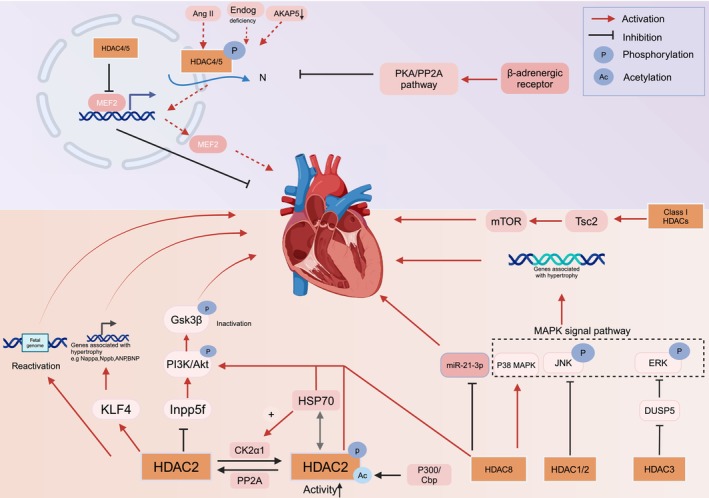
Role of HDAC in myocardial hypertrophy.

Myocardial hypertrophy is an adaptive response of the heart to pressure stimuli, initially serving to counteract the impact of high pressure or volume loads on the ventricular wall. However, when it surpasses the compensatory limit of the myocardium, heart failure can result. Currently, no specific treatment is available for hypertrophic cardiomyopathy or related fibrosis. Therefore, elucidating the mechanisms associated with hypertrophic cardiomyopathy is crucial for improving and preventing the progression of heart failure. Current research suggests that pressure load‐induced myocardial hypertrophy is linked to histone acetylation and that targeting HDAC has the potential to ameliorate myocardial hypertrophy [32]. Currently, there is significant interest in the role of HDAC proteins in the onset and progression of myocardial hypertrophy due to differential subcellular localisation. Class I and Class II HDAC exert distinct effects on cardiac hypertrophy; generally, Class I HDAC functions as prohypertrophic mediators, whereas Class II HDAC acts as antihypertrophic mediators (Figure 1).

HDAC2 is a major regulator of the nuclear distribution of cardiac hypertrophy. Research conducted by Trivedi et al. demonstrated that HDAC2‐knockout (HDAC2‐KO) mice exhibited a limited response to isoproterenol‐induced cardiac hypertrophy and did not display foetal gene reactivation. In contrast, wild‐type mice revealed clear evidence of cardiac hypertrophy [16]. HDAC2 may be involved in the pathogenesis of cardiac hypertrophy via modulation of the PI3K, Akt and GSK3β signalling pathway. The findings indicate that HDAC2 suppresses the transcription of inositol polyphosphate 5‐phosphatase F, a gene responsible for encoding phosphatidylinositol‐4, 5‐bisphosphate and phosphatidylinositol‐3, 4, 5‐triphosphate. This suppression results in enhanced phosphorylation of phosphatidylinositol (3,4,5)‐trisphosphate (PIP3), Akt and GSK3β [16], ultimately leading to inactivation of GSK3β and accelerating the development of cardiac hypertrophy. HDAC inhibitors suppress cardiac hypertrophy by modulating the activity of Kruppel‐like factor 4 (KLF4), which plays a crucial role in the HDAC2‐mediated activation of foetal gene expression associated with hypertrophy under hypertrophic stress [33]. In addition, sphingosine‐1‐phosphate (S1P), an endogenous inhibitor of HDAC, inhibited HDAC2 activity and increased KLF4 expression [34]. Further investigations are warranted to elucidate the specific mechanisms underlying the involvement of S1P and HDAC2 in cardiac hypertrophy through the modulation of HDAC2 activity. In addition, overexpression of HDAC2 in hypertrophic myocardium caused increased susceptibility to ventricular arrhythmias [35]. HDAC2 knockdown increases H3K4me3‐directed Kv channel‐interacting protein 2 (KChIP2) expression through CNOT4‐mediated KDM5 degradation to alleviate ventricular arrhythmias during cardiac hypertrophy [35]. HDAC2 inhibition is a novel therapeutic strategy to prevent cardiac hypertrophy‐related electrophysiological remodelling.

Research has found that acetylation and phosphorylation of HDAC2 are linked to cardiac hypertrophy [36]. Protein phosphatase (PP) 2A can bind HDAC2 and phosphorylate HDAC2 to inactivate it. However, when the heart is subjected to stress such as hypertension or phenylephrine, the interaction between PP2A and HDAC2 is disrupted [36]. At the same time, casein kinase 2α1 (CK2α1) translocates to the nucleus to bind and phosphorylate HDAC2 [37], thereby activating HDAC2 activity. Furthermore, hypertrophic stress induces the p300/CBP‐associated factor (PCAF) to bind specifically to lysine 75 (K75) of HDAC2, catalysing its acetylation [38]. Surprisingly, in HDAC2K75R (acetylation‐resistant HDAC2), the acetylation‐resistant mutant failed to interact with CK2α1, resulting in a significant reduction in S394 phosphorylation and a reduction in HDAC2 intrinsic activity [38]. That is, acetylation of HDAC2 K75 is essential for the phosphorylation interaction with CK2α1.

CK2α phosphorylates HDAC2 to facilitate the interaction between heat shock protein (Hsp70) and HDAC2, while simultaneously promoting positive feedback of HDAC2 phosphorylation [39]. Additionally, Hsp70 is involved in the development of cardiac hypertrophy via the Akt pathway by interacting with HDAC2, which plays a direct role in this process [40]. Hsp70 facilitates the activation of HDAC2 in response to various hypertrophic stress stimuli, thereby contributing to the development of a cardiac hypertrophic phenotype.

Taken together, HDAC2 phosphorylation constitutes a pivotal upstream regulatory mechanism driving the pathogenesis of cardiac hypertrophy. The acetylation of HDAC2 K75 triggered by hypertrophic stress is essential for the phosphorylation of CK2α1, and the phosphorylation and acetylation of HDAC2 interact to dynamically regulate the activity of HDAC2. Following exposure to hypertrophic stimuli, PP2A and CK2α1 form molecular complexes with HDAC2, inducing its site‐specific phosphorylation and consequent enzymatic activation. This activated HDAC2 orchestrates signal transduction through the PIP3/Akt/GSK3β axis, culminating in KLF4‐dependent transcriptional reprogramming that facilitates pathological cardiac remodelling.

Silencing Class I HDAC genes in a mouse model of thoracic aorta constriction‐induced hypertrophic stimulation led to reduced mTOR activity. This is because Class I HDAC inhibits the expression of tuberous sclerosis complex 2 (TSC2), which activates mTOR and contributes to the development of cardiac hypertrophy [41]. Class I HDAC has also been implicated in the pathological progression of cardiac hypertrophy via the MAPK pathway. According to Ferguson et al., HDAC3 suppresses dual‐specificity phosphatase 5 (DUSP5) expression and subsequently inhibits extracellular signal‐regulated kinase (ERK)1/2 phosphorylation [42]. Additionally, HDAC1 and 2 are involved in inhibiting c‐Jun N‐terminal kinase phosphorylation [42].

HDAC8 serves as a mediator in the promotion of myocardial hypertrophy and fibrosis by modulating gene expression and MAPK and Akt signalling pathways, presenting itself as a potential therapeutic target for addressing myocardial hypertrophy. In a mouse model of cardiac hypertrophy and fibrosis, observation was made regarding the upregulation of HDAC8 leading to an increase in the expression of cardiac hypertrophy‐related genes, such as natriuretic peptide A (NPPA), NPPB, atrial natriuretic peptide (ANP) and brain natriuretic peptide (BNP), through the activation of the p38 MAPK signalling pathway. This mechanism plays a crucial role in the progression of cardiac hypertrophy [43]. A previous study conducted by Yan et al. found that HDAC8 facilitates the phosphorylation of Akt and GSK3β and suppresses the expression of miR‐21‐3p, which exerts an inhibitory effect on myocardial hypertrophy [44]. Taken together, these findings suggest that HDAC8 regulates pro‐cardiac hypertrophy.

Collectively, Class I HDACs are capable of modulating the Akt, MAPK and mTOR signalling pathways, as well as the expression of genes associated with hypertrophy, thereby contributing to the initiation and progression of cardiac hypertrophy.

Class II HDAC plays an anti‐hypertrophic role in cardiac hypertrophy, with research mainly focused on HDAC4 and HDAC5. The nuclear shuttling of HDAC4 and HDAC5 is intricately regulated by their phosphorylation levels. The nuclear accumulation of HDAC4 and HDAC5 exerts inhibitory effects on the transcriptional activity of MEF2, thereby contributing to the pathogenesis of cardiac hypertrophy. Additionally, nuclease endonuclease G (Endog) is a key determinant of cardiac hypertrophy. Studies have found that Endog deficiency can promote cardiac hypertrophy through reactive oxygen species (ROS) accumulation, activation of the Akt and GSK3β signalling pathways, and HDAC4 and HDAC5 nuclear export [45]. Angiotensin II (ANG II) enhances the phosphorylation of HDAC4 and HDAC5, leading to their nuclear export and a subsequent increase in histone acetylation. This process upregulates hypertrophy‐related genes and MEF2 transcription, ultimately promoting the development of cardiac hypertrophy [46, 47]. Additionally, down‐regulation of A‐kinase anchoring protein 5 (AKAP5) enhances the activity of CaMKII, HDAC4 and HDAC5, leading to the nuclear export of HDAC4 and HDAC5 and the promotion of cardiomyocyte hypertrophy following hypoxia/reoxygenation (H/R) [48]. The *β*‐adrenergic receptor (*β*‐AR) exerts an inhibitory effect on MEF2 transcription by facilitating the dephosphorylation and nuclear accumulation of HDAC5 through the protein kinase A and PP2A pathway [49]. This regulatory mechanism suggests that *β*‐AR may contribute to the protection against maladaptive myocardial hypertrophy and pathological myocardial remodelling by modulating HDAC5.

In conclusion, HDACs are promising therapeutic targets for treating cardiac hypertrophy. Their involvement in the pathological progression of hypertrophy via deacetylation across various signal transduction pathways has been extensively studied, with a particular emphasis on HDAC2, 4 and 5. Notably, HDAC2 exhibits opposing regulatory roles with HDAC4 and HDAC5. The potential of HDACs in the prevention and treatment of cardiac hypertrophy is substantial, key research priorities into the roles of additional isoforms.

### 4.2 Role of HDAC in Atherosclerosis and Coronary Heart Disease

**FIGURE 2:**
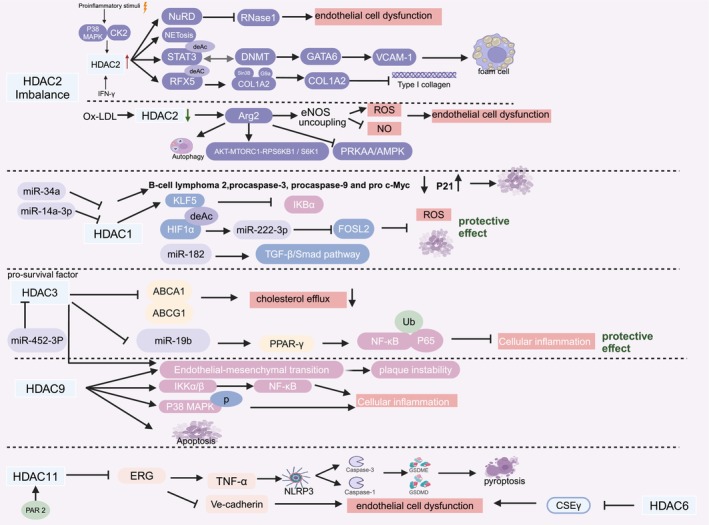
Role of HDAC in atherosclerosis and coronary heart disease.

Despite the declining incidence and mortality rates of ischemic heart disease, atherosclerosis remains a major cause of vascular disease worldwide. Therefore, elucidating the pathogenesis of AS is important [50]. For the study of AS, the role of protein post‐translational modification has gradually attracted attention [51], with acetylation being the most widely studied modification [52]. A variety of HDACs play a role in atherosclerosis and coronary heart disease (Figure 2).

#### 4.2.1 Role of HDAC2 in Atherosclerosis and Coronary Heart Disease

Most current studies have shown that HDAC2 plays a role in foam cell formation and endothelial cell dysfunction, and is involved in the occurrence of AS, which can also lead to increased plaque instability. Hu et al. found that the binding of HDAC1 and HDAC2 to signal transducer and activator of transcription 3 (STAT3) results in its deacetylation and subsequent interaction with DNA methyltransferases, leading to the inhibition of GATA6 promoter demethylation and promotion of GATA6 transcription [53]. Upregulation of GATA6 further enhances the expression of vascular cell adhesion molecule (VCAM‐1) [53], which is known to play a crucial role in foam cell formation and is implicated in the pathogenesis of atherosclerosis. Proinflammatory stimuli in human endothelial cells (HAECs) have been shown to activate p38 MAPK and CK2 activity, leading to the upregulation of HDAC2 expression. This results in the binding of HDAC2 to the nucleosome remodelling and deacetylase (NuRD) complex, which subsequently interacts with endonuclease ribonuclease 1 (RNase1) [54]. Transcription inhibition leads to reduced RNase1 expression and contributes to endothelial dysfunction [54]. Inflammation induced by neutrophil extracellular traps (NET) plays an important role in atherosclerosis progression. Low shear stress promotes NETosis via the Piezo1‐HDAC2 axis [55]. HDAC2 affects atherosclerotic plaque instability. Upon stimulation by the proinflammatory cytokine interferon‐γ, HDAC2‐mediated deacetylation of regulatory factor X 5 recruits Sin3B to the collagen type I α2 chain (COL1A2), which cooperates with G9a to cause COL1A2 transcriptional repression. Inhibition of type I collagen formation is a key process in AS formation [56, 57].

However, opposing views on the role of HDAC2 in AS exist. The study by Hori et al. revealed that HDAC2 overexpression protects against endothelial dysfunction and AS development in mice [58]. Similarly, another study revealed that oxidised low‐density lipoprotein (Ox‐LDL) stimulated human aortic endothelial cells (HAECs), leading to decreased HDAC2 and increased acetylation of the arginase 2 (Arg2) promoter‐stimulated transcription, resulting in the uncoupling of endothelial nitric oxide synthase (eNOS), increased ROS and decreased nitric oxide (NO). This cascade ultimately leads to endothelium‐dependent vasodilation dysfunction, which is an important cause of AS [59]. In apolipoprotein E‐deficient (APOE^−/−^) AS mice, Arg2 impairs endothelial autophagy by regulating mTOR and protein kinase AMP‐activated protein kinase (AMPK) and AMPK signalling pathways in advanced atherosclerosis. Impaired autophagy and mTOR signalling pathways are closely associated with the pathogenesis of atherosclerosis [60]. HDAC2 is a key regulator of endothelial health. In the face of the controversial findings regarding the role of HDAC2 in endothelial cell dysfunction, the use of inhibitors specifically targeting HDAC2 needs to be treated with caution.

#### 4.2.2 Role of HDAC1 in Atherosclerosis and Coronary Heart Disease

The expression of histone deacetylase (HDAC) 1 was reduced in atherosclerotic lesions and HAECs treated with ox‐LDL. Li et al. found that miR‐34a targets HDAC1, downregulates the protein expression of B‐cell lymphoma 2, procaspase‐3, procaspase‐9 and c‐Myc, upregulates the expression of p21, promotes apoptosis of endothelial cells and participates in the occurrence and development of AS [61]. Another study also found that HDAC1 expression was reduced in an animal model of atherosclerosis and found that overexpression of HDAC1 enhanced the effect of miR‐222‐3p targeting Fos‐like antigen 2, thereby inhibiting vascular endothelial cell apoptosis through deacetylating hypoxia‐inducible factor‐1α [62]. HDAC1 expression was reduced in the aortic wall of mice with AS induced by a high‐fat diet (HFD). The miR‐146a‐3p/HDAC1/Krüppel‐like factor 5 (KLF5)/ NF‐κB inhibitor α (IκBα) axis is involved in arterial plaque formation [63]. During the formation of atherosclerotic plaque, HDAC1 expression is inhibited by miRNAs. HDAC1 participates in apoptosis, inflammatory responses and ROS accumulation through a variety of signalling pathways. These findings suggest that the upregulation of HDAC1 has a potential therapeutic role in AS.

HDAC1 is overexpressed in patients with coronary heart disease (CHD). In addition, HDAC1 inhibition of miR‐182 expression in CHD rat cardiomyocytes leads to the activation of transforming growth factor‐*β* and small mothers against the decapentaplegic (SMAD) pathway, which promotes the expression of CHD [64]. This suggests that HDAC1 aggravates CHD. Notably, changes in HDAC1 expression differed between AS formation and CHD, highlighting distinct roles for HDAC1 in these two conditions.

#### 4.2.3 Role of HDAC3 in Atherosclerosis and CHD

HDAC3 is a pro‐survival factor. HDAC3 knockdown has been shown to cause AS and vascular rupture in ^−/−^APOE mice [65]. HDAC3 also increases atherosclerotic plaque instability and inhibits cholesterol efflux from foam cells, which may play a role in the development of atherosclerosis. Chen et al. found that HDAC3 expression was upregulated and endothelial‐to‐mesenchymal transition (EndMT) was exacerbated in APOE^−/−^ mice induced by an HFD. Instability of arterial plaques [66]. LncRNA Kcnq1 overlapping transcript 1(Kcnq1ot1) competitively inhibited the inhibition of HDAC3 by miR‐452‐3p, thereby inhibiting ATP‐binding cassette sub‐family A 1 (ABCA1) transcription and its mediated cholesterol efflux process [67]. Inhibition of HDAC3 increased the gene expression of cholesterol efflux regulators ATP‐binding cassette transporters ABCA1 and ABCG1 and also enhanced the metabolic process of macrophages, which played a protective role in AS [68].

Wang et al. found that the HDAC3 up‐regulation inhibited the gene expression of miR‐19b, upregulated the transcription of peroxisome proliferator‐activated receptor (PPAR‐γ) and inactivated the NF‐κB p65 protein by ubiquitination, thereby inhibiting the inflammatory response and AS progression [69]. However, the expression level of HDAC3 protein decreased in ox‐LDL‐treated human umbilical vein endothelial cells (HUVECs) [69]. This suggests that an increased expression level of HDAC3 in AS is beneficial for suppressing inflammation.

HDAC3 also affects the long‐term prognosis of coronary artery disease (CAD). Lysine methyltransferase SET and MYND domain‐containing protein 2 promotes the transcription of HDAC3, which in turn promotes the expression of serum response factor. This cascade promotes the proliferation of vascular smooth muscle cells and the formation of neointima, thereby increasing the possibility of coronary restenosis and affecting the long‐term prognosis of CHD [70].

In conclusion, HDAC3 plays a role in the pathogenesis of AS, the inflammatory response associated with AS and the long‐term prognosis of CHD. Thus, HDAC3 has great potential as a therapeutic agent.

#### 4.2.4 Role of HDAC9 in Atherosclerosis and CHD

HDAC9 is a key regulator of hypercholesterolemia‐driven vascular inflammation. HDAC9 mediates the deacetylation of IκB kinase α/β (IKKα/β) and promotes the activation of NF‐κB signalling [71]. Thus, HDAC9 drives the inflammatory response of macrophages and endothelial cells and increases the instability of atherosclerotic plaques. Additionally, HDAC9 increased plaque instability by promoting EndMT [72]. Kuang et al. found that HDAC9 could mediate ox‐LDL‐induced inflammatory injury in vascular endothelial cells by regulating the phosphorylation level of p38 MAPK, leading to AS [73]. Additionally, HDAC9 is involved in ox‐LDL‐induced endothelial cell apoptosis and the expression of important epigenetically‐regulated inflammatory mediators [74]. HDAC9 mediates the inflammatory response and endothelial cell dysfunction and increases atherosclerotic plaque instability during the progression of AS through a variety of signalling pathways. Furthermore, the HDAC9 expression level was higher in patients with CAD than in healthy controls, suggesting that HDAC9 expression levels can be used as potential biomarkers for CAD detection.

#### 4.2.5 Role of HDAC11/6 in Atherosclerosis and CHD

Few studies have investigated the mechanism of action of HDAC11, a relatively new HDAC protein, in AS. HDAC11 affects pyroptosis and vascular endothelial barrier function, which may be involved in AS pathogenesis via the pyroptotic pathway. Yao et al. elucidated that the upregulation of HDAC11 expression mediates endothelial pyroptosis in APOE^−/−^ mice fed an HFD. HDAC11 may promote tumour necrosis factor‐alpha (TNF‐α), NLRP3 inflammasome, caspase‐1, gasdermin D (GSDMD) and caspase‐3, and gasdermin E pathways by regulating the E26 transformation‐specific (ETS)‐related gene (ERG) acetylation in HUVECs, leading to pyroptosis [13]. Protease‐activated receptor (PAR) 2 upregulates the expression of HDAC11, which then HDAC11 forms a complex with the Ve‐cadherin transcription factor to inhibit ERG transcription, inhibit VE‐cadherin and impair vascular endothelial barrier function [75]. This suggests that HDAC11 may be involved in the process of PAR2 in promoting atherosclerosis.

HDAC6 is involved in oxLDL‐mediated inhibition of endothelial cystathionine γ‐lyase (CSEγ) transcription and expression in AS cells and animal models. HDAC6 expression is upregulated in AS models [76]. As a specific regulator of CSEγ, HDAC6 contributes to endothelial cell dysfunction and affects vascular homeostasis.

Research on HDAC6 and HDAC11 is relatively limited; however, these two isoforms represent novel therapeutic targets and require additional mechanistic studies.

### 4.3 Role of HDAC in Heart Failure

**FIGURE 3:**
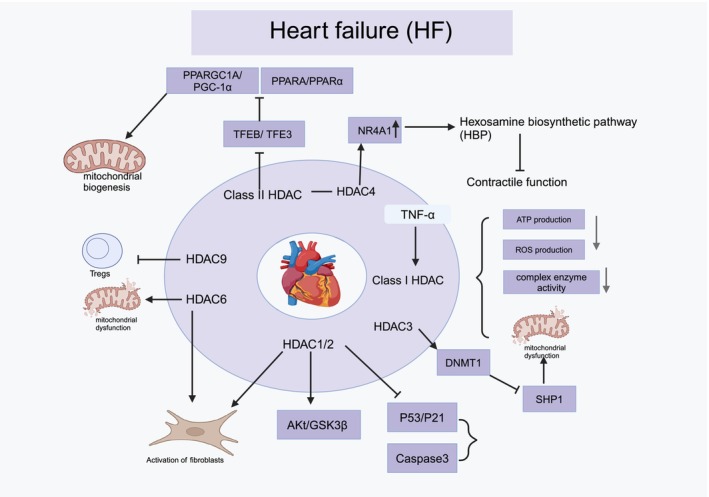
Role of HDAC in heart failure.

Heart failure is a chronic and progressive clinical condition resulting from structural or systolic and diastolic dysfunction, which is categorised into two types: heart failure with reduced ejection fraction and heart failure with preserved ejection fraction (HFpEF) [77]. Owing to cardiac impairment, heart failure results in venous blood stasis and insufficient arterial perfusion, causing circulatory disturbances [78]. The morbidity and mortality rates associated with heart failure remain high, putting considerable pressure on the healthcare system [79]. The role of epigenetics in heart failure has attracted increasing attention, particularly regarding histone post‐translational modifications. Additionally, research has shown that HDAC plays a role in MF, inflammation and oxidative stress related to heart failure and contributes to myocardial remodelling after the condition (Figure 3).

The number of regulatory T cells (Tregs) is reduced in individuals suffering from heart failure, a phenomenon associated with the advancement of the condition. Liao et al. discovered that HDAC9 levels were elevated in patients with heart failure and were directly proportional to the severity of the condition. Furthermore, HDAC9 levels reveal a negative correlation with Tregs and left ventricular ejection fraction [80]. Hence, alterations in HDAC9 expression could serve as potential biomarkers for assessing the severity of heart failure. Further research is warranted to elucidate the molecular mechanisms that link HDAC9 and Tregs. Histone deacetylase 3 increases the level of DNMT1 by deacetylating the DNMT1 ubiquitination pathway, which in turn inhibits the expression of SHP‐1, thereby promoting heart failure [81]. The *N*‐terminal proteolytic derived fragment of HDAC4 (HDAC4‐NT) inhibits the expression of the nuclear orphan receptor Nr4a1, thereby inhibiting the activation of the hexosamine biosynthesis pathway (HBP) and normally regulates the contractile function of cardiomyocytes [82]. Mice with HDAC6 gene deletion exhibit decelerated progression of heart failure characterised by a preserved ejection fraction [83]. This suggests the potential involvement of HDAC6 in myofibroblast activation and mitochondrial function.

The involvement of HDAC in the pathogenesis of heart failure is attributed to their regulation of mitochondrial biogenesis and dysfunction. Both transcription factors (TFEB) and TFE3 upregulate the expression of PPARGC1A (encodes PGC‐1α) and PPARA (encodes PPARα), while both transcription factors are repressed by Class IIa HDACs [84]. PGC‐1α serves as a master regulator in regulating mitochondrial biogenesis. TNF‐α, a proinflammatory factor, has the potential to impair myocardial function through various mechanisms and impact patient prognosis. Furthermore, HDAC is implicated in the induction of mitochondrial dysfunction in response to TNF‐α stimulation. A study conducted by Lkhagva et al. revealed that TNF‐α stimulation led to the upregulation of Class I HDAC activity and expression [85]. Furthermore, HDAC inhibitors were able to restore TNF‐α‐induced mitochondrial dysfunction, including decreased basal respiratory function and ATP synthesis, as well as increased ROS production and abnormal activity of complexes I and II in the mitochondrial electron transport chain [85]. HDAC is involved in TNF‐α‐induced mitochondrial dysfunction and changes in complex enzyme activity in cardiomyocytes, indicating its potential as a target for heart failure. In a mouse model of congestive heart failure, HDAC1 and HDAC2 are upregulated, which promotes myofibroblast activation, Akt/GSK3β signalling, and inhibits Caspase‐3 and p53, and p21 axis [86]. Class I HDAC also plays a role in MF after heart failure and affects prognosis. HDAC inhibitors can improve fibrosis after heart failure [86].

In summary, current research suggests that HDAC is involved in the occurrence and development of heart failure, mainly playing a regulatory role in mitochondrial function, dysfunction and cardiac inflammation. HDAC also represents effective targets for treating heart failure under pathological conditions. However, the molecular mechanisms and modes of action of HDAC in heart failure require further investigation.

### 4.4 Role of HDAC in Myocardial Fibrosis and Remodelling

**FIGURE 4:**
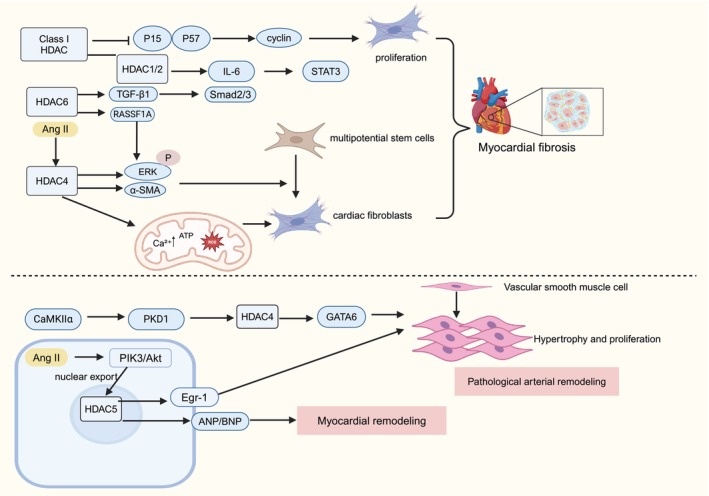
Role of HDAC in myocardial fibrosis and remodelling.

MF is caused by the excessive deposition of cardiac extracellular matrix proteins [87], which leads to heart failure [88] and affects myocardial diastolic function. Advances in epigenetics have brought significant attention to the role of histone modification, particularly histone acetylation modification in MF [89]. Recent studies have shown the involvement of Class I HDAC, HDAC4 and HDAC6 in the pathogenesis of MF (Figure 4).

Class I HDAC are pivotal regulators of cardiac fibrosis and are involved in the promotion of cardiac fibroblast activation. They facilitate the proliferation of cardiac fibroblasts by suppressing the expression of p15 and p57 while inducing cyclin activity [90]. Additionally, HDAC acts as an intermediary mediator in angiotensin II (ANG II)‐induced MF [90, 91]. ANG II stimulates elevated levels of mitochondrial calcium (Ca^2+^), ATP and ROS production through the activation of HDAC activity [4]. This, in turn, activates cardiac fibroblasts and contributes to MF progression. In the ANG II‐induced MF mouse model, the differentiation of mesenchymal stem cells into myofibroblasts is dependent on HDAC4 and requires ERK phosphorylation. Knockdown of HDAC4 inhibited ANG II‐mediated α‐smooth muscle actin (α‐SMA) expression and ERK phosphorylation [92]. In a murine model of congestive heart failure (CHF), HDAC1 and HDAC2 also contributed to MF by participating in the IL‐6/STAT3 signalling pathway [93]. Additionally, HDAC6 is upregulated in isoproterenol‐induced MF tissues, where it exerts inhibitory effects on the Ras association domain family member 1A (RASSF1A), ERK1 and ERK2 signalling pathway, implicating its involvement in pathogenesis [94]. HDAC6 also inhibits myocardial fibrosis and remodelling in mice with myocardial infarction by regulating TGF‐β1/Smad2/3 signalling pathway [95].

In conclusion, HDAC modulated the differentiation of cardiac fibroblasts by regulating cell cycle progression, mitochondrial function, the IL‐6/STAT3 pathway and the ERK pathway. This regulation contributes significantly to the pathogenesis of MF. Given its multifaceted involvement in MF, HDAC is a promising therapeutic target that requires isoform‐specific targeting strategies into its molecular mechanisms.

Myocardial remodelling is a morphological and structural change in the heart that occurs in a variety of heart diseases, such as myocardial infarction, hypertension and diabetic cardiomyopathy. These changes are closely related to patient prognosis [96]. In a mouse model of transverse aortic constriction(TAC), upregulation in the expression of HDAC5 has been observed, which leads to the inhibition of ANP and BNP expression. This inhibition is associated with pathological myocardial remodelling, thereby contributing to cardiac remodelling [97]. Epigallocatechin gallate has been shown to effectively inhibit and delay myocardial remodelling in TAC mice by targeting HDAC5 [97]. Furthermore, HDAC inhibition using Mocetinostat has been shown to ameliorate cardiac remodelling in heart disease [98, 99]. Kim et al. found that CaMKIIα, protein kinase D1, HDAC4 and GATA‐binding factor 6 (GATA6) pathway positively regulated vascular smooth muscle cell (VSMC) hypertrophy and proliferation, resulting in hypertensive pathological arterial remodelling [100]. ANGII‐induced HDAC5 phosphorylation and subsequent nuclear export occur via the PI3K/Akt pathway. Akt regulates ANG II‐induced early growth response protein‐1 (Egr‐1) expression through HDAC5 and participates in VSMC hypertrophy [101]. Additionally, HDAC5 is also involved in pathological arterial remodelling. Targeting HDAC has a great potential for improving CVD outcomes. However, a limited body of research on the mechanism of HDAC in cardiac remodelling exists.

### 4.5 Role of HDAC in Ischemic Reperfusion

I/R refers to myocardial damage caused by the rapid recovery of blood supply to the heart that is rapidly restored after myocardial ischemia.

**FIGURE 5:**
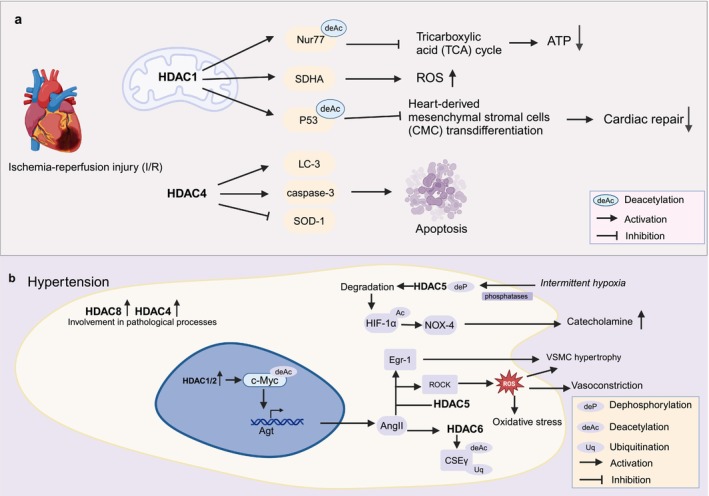
Role of HDAC in ischemic reperfusion and hypertension.

HDAC1 plays a crucial role in mediating the inflammatory response and mitochondrial dysfunction during I/R. I/R‐induced cell injury is a complex process caused by functional metabolic disorders involving multiple signalling pathways. Recent studies have shown that HDACs are involved in the inflammatory response and mitochondrial function during I/R, thereby affecting cardiomyocytes (Figure 5a). However, research in this area remains limited, highlighting the need for further research to clarify the mechanism of I/R and to explore effective treatment aimed at alleviating the mortality associated with myocardial infarction [102].

HDAC1 disrupts the TCA cycle via the deacetylation of Nur77, promoting macrophage inflammation in I/R mice [103]. In a study by Herr et al. using an isolated rat heart model of I/R, HDAC1 was found to be localised in the mitochondria of cardiomyocytes. Mitochondrial HDAC1 was shown to activate succinate dehydrogenase activity and upregulated ROS production during reperfusion, which contributes to the early pathogenesis of I/R [104]. Furthermore, HDAC1‐KO increased the acetylation level of p53, promoting its expression and facilitating cardiac repair mediated by heart‐derived mesenchymal stromal cell transdifferentiation in ischaemic cardiomyopathy [105]. These findings indicate that HDAC1 affects cardiac repair in ischaemic cardiomyopathy.

Overexpression of HDAC4 increases the expression of LC3‐II and caspase‐3, downregulated superoxide dismutase (SOD)‐1, increased I/R injury, reduced infarct size and affected the recovery of ventricular function [106]. Additionally, HDAC4 overexpression increases apoptosis in the ischemic myocardium and exacerbates ventricular dysfunction [25].

In conclusion, HDACs play a pivotal role in I/R injury, mainly by regulating mitochondrial metabolic function and inflammation. Current studies, particularly on HDAC1 and HDAC4, have shown their involvement in exacerbating I/R injury by promoting mitochondrial dysfunction and increased apoptosis. Recent research has also shown that HDAC inhibition can ameliorate damage caused by I/R. Further investigation into the molecular mechanism and pathway through which HDACs influence I/R injury is crucial and could contribute to the development of novel therapeutic strategies aimed at reducing myocardial damage.

### 4.6 Role of HDAC in Hypertension

Hypertension is a condition in which blood pressure within the blood vessels is consistently elevated. According to the World Health Organisation, approximately 1.28 billion adults aged 30–79 years have high blood pressure, with the majority (two‐thirds) living in low–and middle‐income countries. Thus, the medical burden associated with hypertension is extremely high. Although hypertension is a common disease with a high incidence, the role of post‐translational modifications of proteins, especially acetylation, in its pathogenesis has gradually received attention. The mechanisms of action of HDAC family members have also been investigated (Figure 5b).

In hypertension induced by an HFD, HDAC1 and HDAC2 are activated, leading to nuclear accumulation. HDAC1 binds to deacetylated c‐Myc at the renal angiotensinogen (Agt) gene promoter, upregulates Agt and ANG II levels, and participates in the pathogenesis of obesity‐related hypertension [107]. Specific inhibition of HDAC8 can reduce hypertension by inhibiting arterial remodelling, vasoconstriction and inflammation [108], suggesting that HDAC8 is a regulator of hypertension and a potential therapeutic target.

HDAC5 and Rho‐associated protein kinase 2 are upregulated in ANG II‐induced hypertension mice, which affect ROS production and mediate vascular hypertrophy, vasoconstriction and oxidative stress in hypertension [109]. These effects are ameliorated in HDAC5‐knockout mice [109]. Additionally, long‐term intermittent hypoxia (IH) plays a role in the treatment of hypertension through lysine acetylation. In an in vitro rat chromaffin cell model, IH induced dephosphorylation of HDAC5 by PP, especially PP1, leading to HDAC5 degradation [110]. HDAC5 degradation leads to increased acetylation of HIF‐1α to promote its transcriptional expression and then upregulates the expression of nicotinamide adenine dinucleotide phosphate (reduced form) oxidase 4 (NOX4) [110]. This eventually leads to the increase of catecholamine levels and blood pressure and participates in the development of hypertension. NOX‐4 is a significant source of ROS [110]. HDAC5 is an early epigenetic regulator of IH‐induced sympathetic activation and hypertension. HDAC5 also regulates ANG II‐induced Egr‐1 expression and participates in VSMC hypertrophy [101]. Additionally, the study by Kim et al. found that HDAC4 was involved in the regulation of arterial pathological changes during the pathogenesis of hypertension [100]. Class II HDAC is considered a potential therapeutic target for hypertension‐mediated pathological vascular remodelling.

HDAC6 expression is increased in ANG II‐induced hypertension, which leads to the deacetylation and ubiquitination‐mediated degradation of CSEγ, and participates in the occurrence of hypertension [111].

In summary, HDAC5 plays an important role in the early pathogenesis and vascular remodelling of ANG II‐induced hypertension, HDAC6 affects vascular relaxation function, while HDAC1 and HDAC2 regulate the transcription of genes related to blood pressure regulators. The specific molecular pathways involving HDAC deserve further study, and targeting HDACs presents a promising treatment method.

### 4.7 Role of HDAC in Pulmonary Arterial Hypertension

Pulmonary arterial hypertension (PAH) is a progressive, fatal disease caused by vascular inflammation [112], which leads to pulmonary vascular remodelling, increased resistance and, ultimately, right heart failure [113].

Class I HDAC isoforms are significantly dysregulated in human PAH; overexpression is associated with fibroblast activation, and HDAC8 co‐localises with and interacts with expressing α‐SMA to participate in vascular remodelling [114]. Additionally, HDAC2 expression is significantly increased in right ventricular tissue, which causes cardiac hypertrophy [114].

In monocrotaline (MCT)‐induced PAH rats, the upregulation of HDAC1 caused an increase in matrix metalloproteinase‐9 (MMP‐9) and tissue inhibitor of metalloproteinase‐1 (TIMP‐1), and MMP‐2 and TIMP‐2 ratios by inhibiting miR‐34a levels [115]. This promotes the development of PAH and aggravates pulmonary artery remodelling. Additionally, miR‐124 plays an important role in controlling the proliferation, inflammation and metabolic reprogramming of pulmonary artery adventitial fibroblasts [116]. In PAH, the upregulation of HDAC causes chromatin condensation, which reduces the transcription of mature miR‐124 [117], involving PAH development. HDAC1 plays a role in platelet‐derived growth factor‐induced Akt phosphorylation of nuclear cyclin D1 (CycD1) [118], promoting the proliferation and migration of pulmonary artery smooth muscle cells and playing a significant role in vascular remodelling in PAH. In a neonatal mouse hypoxia‐induced pulmonary hypertension (PH) model, Yang et al. found that HDACs regulate insulin‐like growth factor‐1, which activates the Akt signalling pathway, promotes endothelin‐1 expression and contributes to right ventricular hypertrophy and pulmonary vascular remodelling in PAH [119]. HDACs are key regulators in the development of neonatal PAH.

Perivascular inflammation is a major feature of PAH, and HDAC has been found to mediate pulmonary perivascular inflammation. In the MCT‐induced rat PAH model, multiple HDAC isoforms are upregulated, and HDACi can inhibit NOX expression in the pulmonary artery [120]. These findings indicate that HDACs epigenetically regulate chromosome accessibility, induce NOX gene expression in vivo and in vitro and increase ROS levels involved in PAH pathogenesis. Particularly, Class I HDAC3‐mediated histone deacetylation reduces SOD3 expression in pulmonary artery smooth muscle cells (PASmcs), and loss of vascular SOD3 worsens outcomes in animal models of PH [121].

In PAH, activated fibroblasts in the adventitia of the pulmonary artery promote T cell proliferation, activation and polarisation, contributing to the T cell reprogramming into an inflammatory phenotype. This process can be inhibited by the HDAC inhibitor suberoylanilide hydroxamic acid [122]. This finding suggests that HDACs play a crucial role in the activation of T‐cell inflammatory phenotypes by fibroblasts. Chen et al. found upregulation of HDAC expression in peripheral blood mononuclear cells, mediating the reduction of forkhead box (FOX) P3 Tregs and an increase in programmed cell death‐1 signalling in both patients with PAH and animal models [123]. HDAC is the epigenetic regulation of PH, which is mediated by immune dysregulation.

Excessive nuclear accumulation of HDAC4 and HDAC5 results in the transcriptional repression of the transcription factor (MEF2) [124], a key cis‐acting factor that regulates the expression of many transcriptional targets involved in pulmonary vascular homeostasis. The impairment of MEF2 by Class II HDACs leads to excessive cell proliferation, which plays an important role in the pathogenesis of PAH.

**FIGURE 6:**
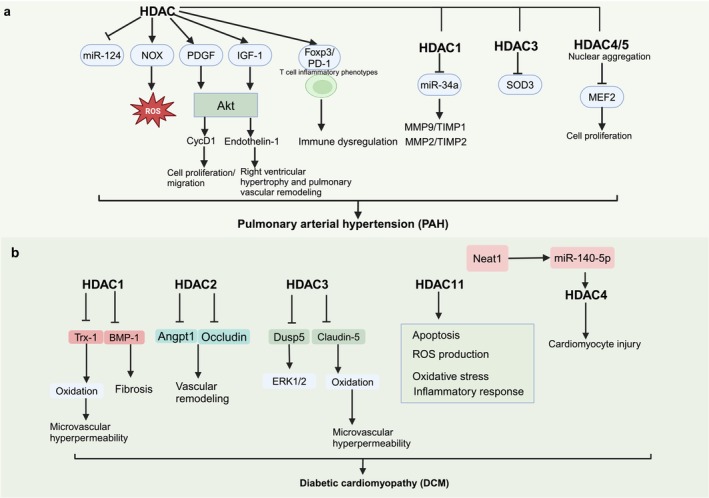
Role of HDAC in pulmonary arterial hypertension and diabetic cardiomyopathy.

Taken together, HDAC plays a role in immune dysregulation, vascular inflammation and pulmonary vascular remodelling caused by abnormal proliferation of endothelial and smooth muscle cells, ultimately leading to right heart failure. The roles of Class I HDAC, Class II HDAC4, and HDAC5 have been studied, proving that epigenetic mechanisms play an important role in PAH. However, the pathogenic role of the HDAC family in PAH needs to be further studied, and the specific downstream targets remain unknown. Clarifying the molecular mechanisms underlying these processes is of great significance for subsequent treatment and prognosis (Figure 6a).

### 4.8 Role of HDAC in Diabetic Cardiomyopathy

DCM is a chronic disease associated with diabetes mellitus, often accompanied by local inflammation, metabolic disorders, oxidative stress, mitochondrial dysfunction, cardiomyocyte apoptosis and neurohumoral dysregulation [125]. Hyperglycaemia is an important risk factor for CVDs. Although the exact pathogenesis of DCM remains unclear, the HDAC protein family plays an important role in the occurrence and development of DCM by regulating protein deacetylation(Figure 6b). Epigenetic mechanisms are involved in the progression of cardiometabolic diseases. Diabetic mellitus rats exhibit hyperglycaemia, HDAC upregulation and heart damage. These findings suggest that histone deacetylation plays a role in DCM [126]. Class I HDAC plays a significant role in DCM initiation, as well as in pathological vascular remodelling and MF. For instance, HDAC1 inhibits the BMP‐7 transcription by enhancing histone deacetylation, thereby promoting MF and aggravating DCM [127]. Overexpression of HDAC2 in diabetic aortic endothelial cells leads to the deacetylation of histone H3 and inhibits the expression of angioprotective genes, such as angiopoietin 1 and occludin, which mediates pathological vascular remodelling in DM [128]. Additionally, diabetes‐induced HDAC3 activation inhibits DUSP5 expression by deacetylating histone H3 in the primer region of DUSP5. This derepression leads to the ERK1 and ERK2 and the initiation of DCM [129]. β‐Hydroxybutyrate can enhance the antioxidant function of cardiomyocytes and antagonise the microvascular hyperpermeability associated with DCM by inhibiting HDAC1 and HDAC3 while promoting the expression of Trx1 and claudin‐5 [130, 131]. β‐Hydroxybutyrate, as a natural inhibitor, shows a significant cardioprotective effect on DCM by inhibiting HDAC1 and HDAC3. Thus, Class I HDAC represents potential targets for the treatment of DCM.

HDAC4‐KO mice developed heart failure in both type 1 and type 2 diabetes models, whereas wild‐type mice did not develop overt signs of heart failure, suggesting that HDAC4 protects diabetic hearts [132]. HDAC4 can either protect or damage the myocardium. O‐GlcNAcylation of HDAC4 at Ser‐642 has been shown to be cardioprotective in diabetes, counteracting pathological Ca^2+^/calmodulin‐dependent protein kinase II signalling [132]. Additionally, catalpol alleviates myocardial injury and plays a cardioprotective effect by regulating the Neat1, miR‐140‐5p and HDAC4 axis in DCM mice [133]. When modifying O‐GlcNAcylation, HDAC4 plays a protective role in the myocardium, whereas unmodified HDAC4 damages DCM cardiomyocytes.

HDAC11 overexpression induces myocardial injury through apoptosis, ROS production, oxidative stress and inflammatory response pathways [14], and contributes to the occurrence and development of DCM.

### 4.9 Role of HDAC in Other Diseases in CVD

#### 4.9.1 Arrhythmia

HDAC downregulation in atrial fibrillation (AF) combined with heart failure or during the early stages of heart failure affects electrophysiological remodelling and may induce arrhythmia. In the early stages of heart failure, decreased HDAC2 expression is associated with delayed repolarisation and potassium channel, inwardly rectifying, subfamily j, member 2 and inwardly rectifying potassium channel 2.1 channel transcription. Specifically, HDAC downregulation is associated with ventricular electrical remodelling and ion channel expression, influencing action potential duration and playing a role in ventricular arrhythmias [134]. HDAC2 is associated with the expression of Ca^2+^‐activated potassium voltage‐gated channel subfamily N (KCNN) potassium channels in patients with AF and heart failure, which promotes the occurrence of atrial arrhythmia. High atrial rates trigger HDAC2‐involved epigenetic remodelling [135, 136]. The inhibition of HDAC reduces the induction of hypertrophy by regulating Ca^2+^ homeostasis [137]. Additionally, the inhibition of Class I HDAC reduces the total duration of AF, atrial fibrosis and infiltration of adipose or immune cells [138]. HDAC inhibitors have shown potential for the treatment of arrhythmia. However, current research on the role of epigenetics in arrhythmia provides a novel direction that may help to further understand the mechanisms underlying AF.

#### 4.9.2 Dilated Cardiomyopathy

Regarding the relationship between HDAC and dilated cardiomyopathy, only HDAC activity in the myocardial interstitial stromal cells of patients with dilated heart disease was found to be 1.5 times higher than that in healthy cells. This increase in HDAC activity was associated with a decrease in mitochondrial membrane potential and ATP levels. However, the location of this connection remains unclear [139].

#### 4.9.3 Myocarditis

Inhibition of HDAC activity enhances myocardial autophagosome formation, which leads to increased coxsackievirus B3 viral replication and, consequently, increased cardiomyocyte apoptosis [140].

#### 4.9.4 Aortic Aneurysm/Dissection (AAD)

HDAC9 is an endogenous risk factor for AAD. Z. Dang et al. showed that HDAC9 inhibited the expression of SOD‐2 and insulin‐like growth factor binding protein‐3, triggered the transformation of VSMC from contractile to synthetic phenotype, increased its proliferation and migration ability and inhibited its apoptosis. It then participated in the occurrence of AAD [141].

#### 4.9.5 Septic Cardiomyopathy

Lipopolysaccharide (LPS) induces the production of TNF‐α in cardiomyocytes, which leads to myocardial depression during sepsis. LPS increases the activity of HDAC3 through mitochondrial reactive oxygen species and c‐Src signalling, inhibits the accumulation of NF‐κB/p65 at the TNF‐α promoter and promotes the expression of TNF‐α, which is involved in the pathogenesis of sepsis‐induced myocardial injury [142]. Cecal ligation and puncture mice exhibit significant myocardial injury, increased HDAC1 and HDAC3 levels, upregulated histone H3 deacetylation levels, activation of the NF‐κB signalling pathway and enhanced inflammatory response [143].

## 5 Overview of HDAC Inhibitors in the Treatment of CVD

HDACs play an important role in the occurrence and development of CVDs and are gradually being considered as therapeutic targets. Recently, HDAC inhibitors have shown promising therapeutic effects in mouse models of AS, PAH, MF, hypertension and other related diseases. The HDAC inhibitors currently approved by the Food and Drug Administration (FDA) include vorinostat, Romidepsin, chidamide, panobinostat and belinostat. An overview of HDAC inhibitors in CVDs is summarised in Table 1. At present, histone deacetylase (HDAC) inhibitors represent the most extensively investigated epigenetic agents in cancer therapeutics. Their mechanisms of action mainly include arresting tumour cell cycle progression, inducing apoptosis of malignant cells, regulating overall immune activity and enhancing tumour killing mediated by natural killer (NK) cells and cytotoxic T lymphocytes. Currently, HDAC inhibitors approved by the U.S. Food and Drug Administration (FDA) are indicated for peripheral T‐cell lymphoma, multiple myeloma, breast cancer and related malignancies. Notably, these compounds also demonstrate therapeutic potential for neurological disorders, particularly Alzheimer's disease and Huntington's disease. Furthermore, emerging evidence suggests their efficacy in managing multisystem pathologies, including chronic kidney disease, chronic obstructive pulmonary disease (COPD), rheumatoid arthritis and hepatic fibrosis.

**TABLE 1:** Overview of HDAC inhibitors in CVD.

Drug	Chemical structure	Target	Targted diseases	References
Mocetinostat	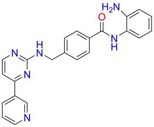	Class I	Myocardial fibrosis, remodelling Ischemic reperfusion injury	[90, 93, 98, 99, 144]
Tubacin	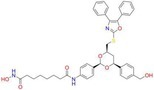	HDAC6	Myocardial fibrosis atherosclerosis hypertension	[76, 94, 111]
Rhein	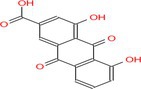		Myocardial fibrosis/remodelling	[145]
Valproic acid	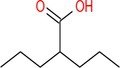	Pan	Myocardial fibrosis/remodelling atherosclerosis Ischemic reperfusion injury hypertension pulmonary arterial hypertension	[73, 92, 146, 147, 148, 149, 150, 151]
TYA‐018	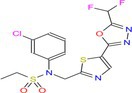		HFpEF	[152]
MPT0E014	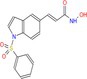		Heart failure diabetic cardiomyopathy arrhythmia dilated cardiomyopathy	[137, 153, 154, 155, 156]
Entinostat	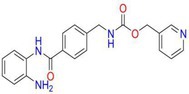	Class I	Heart failure/myocardial fibrosis ischemic reperfusion injury hypertension pulmonary arterial hypertension	[104, 115, 157, 158, 159]
Givinostat	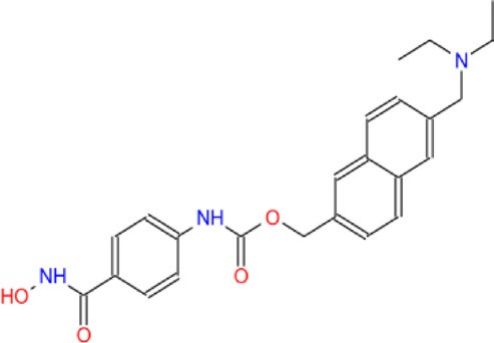	Class I/II	Diastolic dysfunction	[160]
Trichostatin A	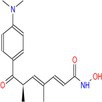	Pan	Atherosclerosis myocardial fibrosis	[91, 161, 162, 163]
Butyrate	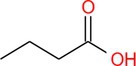	Class I/II	Atherosclerosis pulmonary arterial hypertension myocardial infarction diabetic cardiomyopathy	[164, 165, 166, 167]
Romidepsin	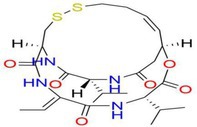	Class I	Atherosclerosis hypertension	[53, 107]
RGFP966	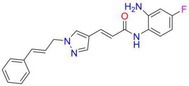	HDAC3	Atherosclerosis hypertension diabetic cardiomyopathy	[66, 129, 159]
Vorinostat	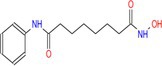	Pan	Atherosclerosis myocardial ischemia hypertension diabetic cardiomyopathy dilated cardiomyopathy	[3, 139, 168, 169, 170, 171]
CG200745	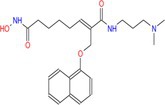	Class I/II	Hypertension, myocardial fibrosis hypertrophy	[172, 173, 174]
PCI34051	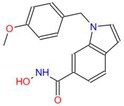	HDAC8	Hypertension	[108]
MC1568	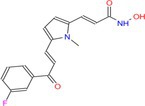	Class II	Hypertension	[100]
YPX‐C‐05	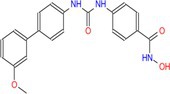	Pan	Hypertension	[175]
Honokiol	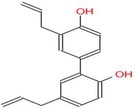	Class I	Hypertension	[176]
Apicidin	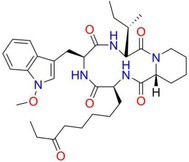	Class I/II	Pulmonary arterial hypertension	[177]
LMK235	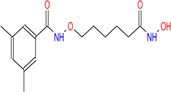	Class I	Hypertension	[178]
Melatonin	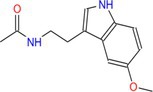	Class I	DEX‐induced programmed hypertension	[179]

*Vorinostat* was first approved by the FDA in 2006 for the treatment of cutaneous T‐cell lymphoma. Recent studies have found that vorinostat has a significant effect on CVD by reducing NOX1, NOX2 and NOX4 transcript levels and reduced the progression of atherosclerosis in ApoE^−/−^ mice by negatively regulating NOX expression and inhibiting the inflammatory response [168]. Additionally, vorinostat induces cardiomyocyte autophagy, which reduces I/R injury during reperfusion [180, 181]. Yang et al. found that vorinostat induced PGC‐1α gene expression, exerted mitochondrial protection, reduced ROS production and prevented myocardial apoptosis [3]. Vorinostat can promote the transcription of Wnt‐induced secretory protein‐1 and its downstream signalling transduction after myocardial infarction [182] and also induce angiogenesis, which is beneficial for the treatment of myocardial infarction. In addition, a study by Kimbrough et al. found that vorinostat promoted the early phenotypic conversion of reparative CD45, CD11b and CD206 macrophages in the post‐myocardial infarction heart. This effect was associated with improved ventricular function and remodelling [169].

Vorinostat can reduce cellular oxidation and restore myocardial contractility in an early diabetic rat model [170]. Vorinostat has recently been found to be a promising treatment for dilated cardiomyopathy; Miksiunas et al. found that vorinostat can improve cardiomyogenic differentiation and enhance mitochondrial energy status, resulting in improved cardiac tissue regeneration capacity [139, 171]. In summary, vorinostat has shown great potential for the treatment of various CVDs, although current studies are mainly at the animal level. Further studies are needed to prove its efficacy and to carry out corresponding clinical studies.

*Valproic acid (VPA)* upregulates SOD1, plays an antioxidant role, reverses acute myocardial infarction‐induced endothelial dysfunction, increases vascular smooth muscle NO bioavailability and aortic blood flow and improves myocardial I/R injury [146]. VPA mitigates inflammatory injury to vascular endothelial cells and plays a protective role in ischemic stroke induced by atherosclerosis [73]. In a rat model of PH, Lan et al. found that pre‐VPA treatment prevented and partially reversed the development of severe PH and reduced inflammation and proliferation in remodelling pulmonary arteries [147]. Furthermore, VPA prevents HFD‐induced hypertension by inhibiting HDAC1 and downregulating ANG II and its receptor, thus providing a new therapeutic option for HFD‐induced hypertension [148]. VPA attenuates cardiac hypertrophy and fibrosis by acetylating mineralocorticoid receptors in hypertensive rats and improves hypertensive prognosis [149, 150, 183]. Additionally, VPA inhibits ANG II‐induced pericyte‐myofibroblast transdifferentiation through MAPK and ERK pathways and delays cardiac fibrosis, which is a promising therapeutic agent for MF [92]. VPA is clinically available, well tolerated and has the potential to delay the development of atrial remodelling and the onset of atrial fibrillation in high‐risk patients [151]. VPA attenuates sepsis‐induced myocardial dysfunction by accelerating myocardial autophagy, increasing phosphatase and tensin homologue expression and inhibiting the Akt and mTOR pathways [184]. In conclusion, VPA demonstrates broad therapeutic efficacy against atherosclerosis, ischaemic myocardial injury, hypertension, PAH and associated fibrotic remodelling.

*Mocetinostat (MGCD0103)*, a novel HDAC inhibitor discovered in 2007, can reduce the proliferation of tumour cells and has good selectivity for HDAC1 [185]. Preclinical studies of mocetinostat in melanoma (NCT03565406), lymphoma (NCT00358982), non‐small cell lung cancer (NCT02805660) and other tumours have been performed in phase II clinical trials; however, no clinical studies on CVD have been conducted to date. Selective inhibition of Class I HDAC by mocetinostat can effectively stop ANG II from causing heart fibrosis in a targeted manner, making it a useful treatment for controlling harmful heart fibrosis [90]. Mocetinostat exerts anti‐fibrosis effects by inhibiting the IL‐6 and STAT3 signalling pathways in CHF cardiac fibroblasts [93]. Additionally, mocetinostat activates cyclic AMP response element‐binding protein and PGC‐1α signalling pathway during I/R in vivo and in vitro, which may alleviate myocardial reperfusion injury and play a cardioprotective role [144]. MGCD0103 can attenuate aortic remodelling in TAC‐induced pressure overload rats by inhibiting aortic wall fibrosis and increasing ANG receptor expression. This may serve as a potential therapeutic target for anti‐aortic remodelling in pressure overload‐induced hypertension‐related diseases [98, 99]. Both in vivo and in vitro investigations of CVDs have revealed that mocetinostat possesses anti‐fibrotic properties, protects cardiomyocytes from I/R injury and retards the pathological remodelling of the aorta.

*MPT0E014* was initially found to have potent anti‐tumour cell proliferative activity [186]; however, an increasing number of studies have also found that it has the potential to treat CVD. MPT0E014 can down‐regulate the expression of IL‐6, p22, SMAD2 and SMAD3, extracellular signal‐regulated kinase 1/2, PPAR isoforms and circulating tumour growth factor‐β1. Additionally, it reduces metabolic abnormalities and the inflammatory response associated with heart failure, thereby improving heart function [153]. MPT0E014 improves cardiac function in diabetic rats by regulating myocardial autophagy, inflammation and insulin signalling [154, 155]. MPT0E014 can also prevent the occurrence and duration of arrhythmia, especially atrial fibrillation, by reducing Ca^2+^ transient amplitude, sodium, Ca^2+^ exchange current and ryanodine receptor expression in cardiomyocytes, thereby regulating Ca^2+^ homeostasis [137]. MPT0E014 inhibits the migration and proliferation of new fibroblasts in isoproterenol‐induced dilated cardiomyopathy, plays an anti‐fibrosis role, improves cardiac contractility and has therapeutic potential for dilated heart disease and heart failure [156]. As a novel HDAC inhibitor, MPT0E014 is mainly used in the study of CVD and currently shows therapeutic prospects for MF, arrhythmia and heart failure. Therefore, more accurate animal experiments and clinical studies are warranted.

*Entinostat* is a benzenamide‐derived HDAC inhibitor synthesised in 1999 that inhibits tumour cell proliferation by affecting the cell cycle [187]. Entinostat has been shown to reduce the prolonged repolarisation associated with heart failure, partially restore KCNH2 and Cav1.3 expression and improve the cardiac electrophysiological remodelling associated with heart failure [157, 188]. In addition, entinostat exhibited anti‐fibrotic effects in both in vivo and in vitro experimental models [157]. Entinostat promotes FOXO3a transcription factor aggregation, induces mitochondrial SOD2 and catalase protein transcription, significantly reduces infarct size and ameliorates I/R injury [104, 158]. Entinostat can restore the level of NO mediated by ANG II, reduce the infiltration of proinflammatory factors and macrophages and play a role in the treatment of hypertension by regulating vascular remodelling, vasoconstriction and the inflammatory response [159]. Through inhibiting HDAC1, entinostat alleviates pulmonary artery remodelling and PAH by upregulating miR‐34a levels, which subsequently reduce MMP‐9 and TIMP‐1 and MMP‐2 and TIMP‐2 levels [115].

In summary, entinostat lowers blood pressure, ameliorates I/R injury, delays non‐vascular remodelling, improves heart failure prognosis in PAH and prevents fibrosis.

*Butyrate*, a short‐chain fatty acid, inhibits HDAC and plays a therapeutic role in the prognosis of atherosclerosis, PH, diabetic cardiomyopathy and myocardial infarction. In the ApoE^−/−^ mice, butyrate can ameliorate atherosclerotic inflammation by regulating macrophage polarisation via the G‐protein coupled receptor 43 and HDAC‐miRNA axis [164]. Chen et al. found that butyrate reduced interstitial fibrosis and active caspase‐3, and that apoptotic staining mediated the reduction in cardiac hypertrophy in mice with diabetic cardiomyopathy in vivo [165]. Butyrate reduces the expression of the pro‐hypertrophic HDACs such as HDAC2 without changing the expression of the anti‐hypertrophic HDAC5 and exhibits beneficial effects on cardiac hypertrophy by reducing collagen levels, improving mitochondrial DNA concentration and preserving left ventricular systolic and diastolic function [189]. In addition, glucose transporters 1 and 4 (GLUT1/4) were upregulated after HDAC inhibition, along with increased GLUT1 acetylation, P38 phosphorylation and myocardial SOD [165]. These findings of manifestations fully demonstrate the role of butyrate as an HDAC inhibitor in improving cardiac function and inhibiting myocardial remodelling in diabetic cardiomyopathy. Butyrate exerts a protective effect against PH by inhibiting macrophage aggregation and proinflammatory factor secretion, with high doses capable of reversing right ventricular hypertrophy [166]. Additionally, butyrate improves tissue repair after myocardial infarction in rats [167]. However, the role of butyrate in the treatment of CVD requires further investigation, and clinical trials are necessary to confirm its efficacy in humans.

*Trichostatin* ***A*** targeted the CCAAT (C), EBPα and PPARγ axis to increase the formation of PPARγ and its downstream targets, ABCA1 and ABCG1. This action inhibits foam cell formation and reduces the induction of inflammatory factors such as TNFα and IL‐1β, which have therapeutic potential in delaying the progression of AS and related CVDs [161]. Trichostatin A (TSA) protects dendritic cells (DC) from oxygen–glucose deprivation and promotes DC migration through serine/arginine‐rich splicing factor 3, pyruvate kinase muscle isozyme 2 and the glycolytic pathway [162], which increases DC infiltration in the infarct area after myocardial infarction. TSA treatment results in increased phosphorylation or acetylation of mitogen‐activated protein kinase 3 (MKK3) in the myocardium, and the activation of the MKK3 and Akt‐1 pathways exerts cardioprotective effects after I/R [163]. Furthermore, TSA inhibits ANG‐II‐induced transcription of MMP9 and IL‐18 by blocking the binding of NF‐κB and AP‐1 to their respective promoter regions [91]. By inhibiting Sp1 binding to the reversion‐inducing cysteine‐rich protein with kazal motifs (RECK) promoter, TSA reversed ANG‐II‐induced RECK inhibition, collagen and fibronectin expression, and cardiac fibroblast migration and proliferation [91]. TSA has anti‐fibrotic and anti‐inflammatory effects on the heart. TSA dose‐dependently inhibited the induction of Nppa, Nppb and the myosin heavy chain (MYH) 7 and MYH6 ratio, resulting in basal hypertrophy‐promoting expression of pathological genes [190]. The effects of TSA on phenylephrine‐induced cardiomyocyte hypertrophy are also noteworthy.

*Romidepsin* is a drug widely used in the treatment of peripheral T‐cell lymphomas, which was approved by the FDA in November 2009 for the treatment of cutaneous T‐cell lymphoma [191]. Romidepsin significantly attenuated TNFα‐induced HAEC surface VCAM‐1 expression and monocyte adhesion by inhibiting HDAC1 and HDAC2 targeting STAT3, GATA6 and VCAM‐1. Additionally, romidepsin reduced the development of diet‐induced atherosclerotic lesions in ApoE mice [53]. Romidepsin treatment abolishes HFD‐induced blood pressure elevation and blocks the upregulation of renal Agt mRNA, proteins, ANG II and serum ANG II [107]. Therefore, romidepsin may have a therapeutic role in atherosclerosis and hypertension.

Some specific HDAC inhibitors, as well as newly developed HDAC inhibitors in ongoing studies, have shown potential applications in the treatment of hypertension, PH and other CVDs. The studies involving these drugs are reviewed below. Current studies on HDAC inhibitors for the treatment of CVDs indicate that HDAC3, HDAC6, HDAC8 and HDAC9 are primarily targeted isoforms. HDAC6‐selective inhibitors—including Tubacin, TYA‐018, LMK235 and Honokiol—have demonstrated promising efficacy in managing hypertension, atherosclerosis and myocardial fibrosis. Compared to pan‐HDAC inhibitors, these compounds exhibit enhanced safety and potency. The HDAC3‐selective inhibitor RGFP966 attenuates atherosclerosis by suppressing EndMT while also showing therapeutic potential for hypertension and DCM. Similarly, the HDAC8‐selective inhibitor PCI34051 effectively mitigates hypertension and inhibits proinflammatory responses. In summary, isoform‐selective HDAC inhibitors represent a promising therapeutic strategy for CVD due to their improved safety profile and target‐specific mechanisms.

Isoforms such as HDAC1 and HDAC2 play indispensable roles in maintaining physiological homeostasis of the circulatory system. However, pan‐HDAC inhibitors may disrupt normal HDAC‐mediated regulatory pathways, leading to off‐target adverse effects. In contrast, isoform‐selective inhibitors enable precise targeting of disease‐associated HDAC subtypes, thereby reducing unintended pharmacological consequences—though their long‐term clinical safety profiles require further validation. Furthermore, given that the pathogenesis of CVD involves dynamic protein–protein interactions and that HDACs can form protein complexes to exert pathological effects, a comprehensive study of the efficacy, target‐binding specificity and pharmacokinetic behaviour of selective HDAC inhibitors remains warranted.

*Tubacin* is an HDAC6 inhibitor that was developed in 2003 [192]. Tubacin can block oxLDL‐mediated reduction of CSEγ expression and CSEγ promoter activity in endothelial cells, thereby increasing CSEγ expression, improving endothelial function, alleviating vasoconstriction and preventing or reversing the development of atherosclerosis and hypertension [76, 111]. Additionally, Tubacin inhibits HDAC6 and promotes the expression of RASSF1A, which plays an anti‐fibrotic role [94]. Tubacin is a potential drug for the treatment of hypertension, atherosclerosis and cardiac fibrosis. *TYA‐018* also is an HDAC6‐specific inhibitor. A study conducted by Ranjbarvaziri et al. revealed that TYA‐018 restored the expression of genes related to hypertrophy, fibrosis and mitochondrial energy production in HFpEF heart tissue [152]. Additionally, TYA‐018 inhibited the activation of human cardiac fibroblasts and enhanced mitochondrial respiration in cardiomyocytes [152]. In conclusion, TYA‐018 targeting HDAC6 has promising applications in HFpEFs. ***LMK235*** is a Class I HDAC6‐preferred HDAC inhibitor that reduces hypertension by inhibiting vasoconstriction and vascular hypertrophy. Choi et al. found that LMK235‐induced vasodilation by promoting NO expression inhibited the increase in aortic wall thickness, decreased CaMKIIα associated with vascular smooth muscle proliferation, reduced ANG II‐induced expression of CycD1 and E2F3 and restored P21 expression [178]. *Honokiol* ameliorates ANGII‐induced hypertension and endothelial dysfunction by inhibiting HDAC6‐mediated cystathionine γ‐lyase degradation [176].

*The research group for functional proteomics 966 (RGFP966)* is an HDAC3‐specific inhibitor. RGFP966 alleviates atherosclerotic lesions in ApoE mice by inhibiting EndMT in atherosclerotic plaques and controlling endothelial cell inflammation [66]. RGFP966 exhibits effects similar to those of entinostat, which relaxes the aorta, reduces blood pressure and reduces macrophage infiltration and inflammatory response induced by ANG II, thus playing an effective role in hypertension [159]. RGFP966 has potential applications in preventing DCM by up‐regulating the DUSP5‐ERK1 and ERK 2 pathways [129]. HDAC3 inhibition has a potential therapeutic value for AS, DCM and hypertension. The development of targeted specific HDAC subtypes can have similar efficacy with higher safety, warranting further study.

*Givinostat* was first approved in the United States of America on 21 March 2024, for the treatment of Duchenne muscular dystrophy in patients 6 years of age or older [193]. Givinostat restores diastolic dysfunction and inhibits cardiac fibroblast activation by attenuating the recruitment of the profibrotic chromatin‐reading protein bromodomain‐containing protein 4 to key gene regulatory elements [194]. This HDAC inhibitor givinostat can enhance myocardial fibril relaxation and is promising for treating human HFpEF [160].

*CG200745 (CG)*, an HDAC inhibitor, inhibited mTORC1 signalling by up‐regulating C, EBP‐β and TSC2 pathways to improve cardiac hypertrophy [172]. Additionally, CG ameliorated HFD‐induced hypertension, as well as subsequent cardiac hypertrophy and fibrosis by inhibiting the HDAC, ANG II and vasoconstrictor axis [173, 174].

*MC1568* can inhibit HDAC II to negatively regulate VSMC hypertrophy and proliferation through CaMKIIα, protein kinase D1, HDAC4 and GATA6 pathways, thereby reducing hypertension [100].

*PCI34051*, an HDAC8‐specific inhibitor, reduces systolic blood pressure by downregulating the mRNA expression of ANGII receptor type 1 and alleviates vascular hypertrophy by reducing the mRNA expression of E2F3 and GATA6 [108]. PCI34051 increased NO vasodilation in HUVECs and decreased the expression of the inflammatory and adhesion molecules VCAM‐1 and intercellular adhesion molecule‐1 in the aortae of ANG II‐treated mice [108].

*YPX‐C‐05*, a novel HDAC inhibitor based on hydroxamic acid, exerts significant antihypertensive and vasodilatory effects through the PI3K, Akt and eNOS pathway and has a promising antihypertensive application [175].

*Rhein* acts as an inhibitor of HDAC Class I and II. Rhein inhibits collagen contraction, suggesting anti‐fibrotic properties in cardiac remodelling, which is associated with HDAC‐dependent protein stabilisation of P53 [145].

*Apicidin* inhibits proliferation, induces cell cycle arrest in neonatal PASMCs, inhibits serum‐induced cell migration and regulates the expression profile of genes encoding prooxidant and antioxidant enzymes [177]. Additionally, apicidin delays PH progression of PH by regulating the phenotype of PASmcs.

*Melatonin* is an antioxidant. Studies have found that melatonin can inhibit HDACs and provide a long‐term protective effect against neonatal dexmethasone‐induced hypertension, similar to TSA [179].

*Emodin* partially blocks pathological cardiac hypertrophy by inhibiting changes in HDAC‐dependent gene expression [195, 196].

The approved HDAC inhibitors are mainly used to treat cancer, but their cardiotoxicity limits clinical use. The application of HDACi in CVDs is mainly in the preclinical stage and there are not enough studies on its side effects and long‐term side effects. HDAC inhibitors cause QT interval prolongation and ventricular arrhythmias, and their cardiac toxicity limits their use in CVD [197, 198]. Pan‐HDAC inhibitors (such as TSA, vorinostat and romidepsin) reduce gap junction conductance, which may increase the occurrence of triggering activity by limiting electrotonic inhibition, combined with sodium current reduction, slow myocardial conduction and increased susceptibility to re‐entry arrhythmias [199, 200]. The study of J. Marlowe et al. showed that HDAC inhibitors do not affect cardiac function by directly blocking ion channels, but by affecting the transcription of ion channel transport proteins and localisation protein genes to the sarcolemmal, inducing prolonged cardiac repolarisation [201]. In addition, they can induce defects in the transport of intracellular potassium channels and inhibit the glycosylation of potassium channels expressed in cells [201]. The prolongation of the QTc interval may be related to the drug concentration in myocardial tissue and HDAC activity [201]. In cancer patients receiving romidepsin therapy, no significant prolongation of the QT interval was observed, although transient tachycardia may occur as a potential cardiovascular effect [202]. Atrial tissues from patients with atrial fibrillation (AF) exhibited a significant increase in HDAC6 activity [203]. Notably, administration of the HDAC6‐selective inhibitor TSA ameliorated AF‐induced electrophysiological abnormalities in vivo [203, 204]. This observation implies that distinct subtypes of HDAC inhibitors exert differential effects on the cardiac conduction system. Notably, isoform‐selective HDAC inhibitors demonstrate dual advantages: they exhibit a lower propensity to induce arrhythmogenic adverse effects compared to pan‐HDAC inhibitors, while concurrently retaining therapeutic efficacy against cardiac electrical abnormalities.

Class I HDACi may aggravate vascular injury during the treatment of CVD. Histone acetylation is also increased in endothelial cells End1 expression, thereby increasing vascular tone; in addition, histone acetylation can increase the expression of proinflammatory genes in VSMCs, which contribute to atherosclerosis, and promote the development of vascular calcification [6]. Exposure to VPA during pregnancy causes CHD. VPA can inhibit HDAC activity, especially HDAC3, and reduce planar cell polarity key genes, Vangl2 and Scrib, expression in cardiomyocytes both in vitro and in vivo, resulting in VPA‐induced abnormal heart development [205]. Therefore, when developing HDAC inhibitors to treat CVD, attention should be paid to their side effects, especially cardiac toxicity and life‐threatening arrhythmias. Caution should also be exercised when administering VPA to young children, especially those with congenital heart disease.

In response to the cardiotoxicity of HDACi, A. J. P. Teunissen et al. developed a nano‐liposome loaded with the HDAC9 specific inhibitor TMP195 (TMP195‐NB), which reduced the overall drug dosage and had significant efficacy [206]. Novel targeted delivery systems combined with nanoparticles provide new directions for the clinical application of non‐HDAC inhibitors. Given the limited clinical efficacy, toxicity, selectivity and other challenges of inhibitors targeting epigenetics, at present, targeted protein degradation (TPD) technology, such as PROTAC, molecular glue and hydrophobic tagging (HyT), has greatly improved the limitations of traditional drugs [207]. Nexturastat A is a highly selective and potent HDAC6 degrader that has shown anti‐inflammatory and anticancer activity in preclinical studies [208]. In addition, PROTACs targeting HDAC3, HDAC4, HDAC8 and Class I HDACs have been developed that can act with high efficiency at nanomolar‐range efficacy, providing a more efficient alternative. The development of molecular glues that are more potent, lighter and have a higher potency than PROTAC for the first time in 2023 may provide a promising avenue for the development of degraders targeting HDAC. Therapeutic approaches targeting HDAC degraders may open new avenues for the treatment of CVDs.

## 6 Conclusion & Future Perspectives

Recent studies have elucidated the role of HDAC in the regulation of cardiovascular physiology and pathology and have established the therapeutic and preventive value of targeting HDAC activity. In general, HDAC inhibition has a positive effect on the cardiovascular system; however, the cardiotoxicity of pan‐HDAC inhibitors has been reported in some clinical trials of cancer, suggesting that caution is warranted in their use. In contrast, an imbalance in HDAC levels contributes to our susceptibility to CVDs such as atherosclerosis, ischemic injury, cardiac fibrosis and hypertrophic cardiomyopathy. Interestingly, Class I and II HDACs exhibit opposing effects in hypertrophic cardiomyopathy. Therefore, the role of HDAC in CVD is not only a simple upregulation to promote disease progression; however, more mechanisms that warrant further exploration are involved. The different mechanisms of the HDAC subtypes need to be further studied. As discussed in this review, several associations between HDAC and CVD provide pharmacological evidence that warrants further development of clinical trials. Additionally, more types of HDAC inhibitors still have potential for development, particularly HDAC‐selective inhibitors, in the context of CVD. Moreover, the side effects of HDAC inhibitors in the treatment of CVD also need to be studied. In addition, targeted delivery systems combined with nanoparticles, HDAC degraders and molecular glues have opened new avenues for the clinical application of HDAC inhibitors. Therefore, systematic exploration, development and testing of the role of HDACs in CVDs, along with the development of selective drugs targeting HDACs, represent our future directions.

## Author Contributions

**Tie‐Ning Zhang and Ni Yang:** conceptualisation, formal analysis, investigation, methodology and original draft preparation. **Li‐Ying Zhang, Yue‐Yue Wang** and **Ri Wen:** methodology, original draft preparation, validation and visualisation.

## Conflicts of Interest

The authors declare no conflicts of interest.

## Data Availability

The data that support the findings of this study are available on request from the corresponding author. The data are not publicly available due to privacy or ethical restrictions.
